# Natural thioallyl compounds increase oxidative stress resistance and lifespan in *Caenorhabditis elegans* by modulating SKN-1/Nrf

**DOI:** 10.1038/srep21611

**Published:** 2016-02-22

**Authors:** Takahiro Ogawa, Yukihiro Kodera, Dai Hirata, T. Keith Blackwell, Masaki Mizunuma

**Affiliations:** 1 Department of Molecular Biotechnology, Graduate School of Advanced Sciences of Matter, Hiroshima University, Higashi-Hiroshima 739-8530, Japan; 2 Drug Discovery Laboratory, Wakunaga Pharmaceutical Co., Ltd, Hiroshima 739-1195, Japan; 3 Joslin Diabetes Center, Harvard Stem Cell Institute, and Harvard Medical School Department of Genetics, Boston, MA 02215, USA

## Abstract

Identification of biologically active natural compounds that promote health and longevity, and understanding how they act, will provide insights into aging and metabolism, and strategies for developing agents that prevent chronic disease. The garlic-derived thioallyl compounds *S*-allylcysteine (SAC) and *S*-allylmercaptocysteine (SAMC) have been shown to have multiple biological activities. Here we show that SAC and SAMC increase lifespan and stress resistance in *Caenorhabditis elegans* and reduce accumulation of reactive oxygen species (ROS). These compounds do not appear to activate DAF-16 (FOXO orthologue) or mimic dietary restriction (DR) effects, but selectively induce SKN-1 (Nrf1/2/3 orthologue) targets involved in oxidative stress defense. Interestingly, their treatments do not facilitate SKN-1 nuclear accumulation, but slightly increased intracellular SKN-1 levels. Our data also indicate that thioallyl structure and the number of sulfur atoms are important for SKN-1 target induction. Our results indicate that SAC and SAMC may serve as potential agents that slow aging.

The human body is constantly exposed to reactive oxygen species (ROS), which are generated by aerobic respiration in the mitochondria and as byproducts of diverse metabolic reactions in cells. Overproduction of ROS causes damage to cellular proteins, lipids and DNA, eventually contributing to various chronic diseases including cancer, diabetes, Parkinson’s and Alzheimer’s disease, cardiovascular disease and chronic inflammation^1^. Therefore, cumulative oxidative damage to the cells may also influence aging. It is known that antioxidant vitamins C and E existing in a wide variety of foods act cooperatively to protect cells from lipid peroxidation by directly neutralizing harmful hydroxyl radicals^2^. Additionaly, sulforaphane, a natural dietary isothiocyanate produced in cruciferous vegetables such as broccoli and broccoli sprouts, has been shown to induce phase II detoxification genes, e.g. Heme oxygenase-1 (HO-1), NAD(P)H: quinone oxidoreductase, γ-glutamylcysteine synthetase and glutathione *S*-transferases (GSTs), through activating Nrf2 (NF-E2-related factor) signaling^3^. The induction of these enzymes by sulforaphane protects cells from damage associated to oxidative stress in diverse *in vivo* and *in vitro* experimental conditions^3^. Therefore, intake of these natural compounds through diet could help to prevent pathogenesis of chronic diseases and contribute to slow aging, or in other words extend health span of organisms.

Garlic (*Allium sativum* L.) has been widely used as a food and folk medicine since ancient times. A number of studies have indicated that garlic possesses diverse pharmacological potentials related to chronic diseases, such as anticancer^4^, antithrombotic^5^, hypolipidemic^6^ and hepatoprotective activity^7^. Many of these beneficial effects have been shown to be attributed to garlic characteristic organosulfur compounds (OSC), including *S*-allylcysteine (SAC) and *S*-allylmercaptocysteine (SAMC)^8^,^9^,^10^,^11^,^12^,^13^. SAC and SAMC are the major water-soluble OSCs naturally occurring during aging process of garlic, and known to act as free radical scavengers^14^. Therefore, some of these protective effects of SAC and SAMC could potentially be explained by their radical scavenging activity. While some studies have demonstrated that SAC and SAMC inhibited growth of human cancer cells *in vitro*^12^,^15^, and development of chemically induced cancers or growth of implanted tumors *in vivo* along with increasing levels of GSTs^8^,^16^. GSTs play a key role in the phase II detoxification response, which provides a conserved defense against oxidative stress^17^. More recent study demonstrated that SAC treatment protected primary cultured neurons and mice against oxidative insults and middle cerebral artery occlusion-induced ischemic damages, respectively, through increases in the levels of Nrf2 protein and target genes expressions, such as γ-glutamylcysteine synthetase catalytic subunit (GCLC), γ-glutamylcysteine synthetase modulatory subunit (GCLM) and HO-1^18^. Because development of cancer, oxidative stress response and apoptosis are strongly associated with aging, we considered the question of whether SAC and SAMC can retard aging. However, the ability of SAC and SAMC to modulate organismal aging and the potential mechanisms involved have not been reported.

Since the finding in *Caenorhabditis elegans* that reduction in signaling through the conserved insulin/IGF-I signaling (IIS) pathway results in more than double the mean lifespan compared with wild-type^19^, aging has become a particularly active area of research. Further studies have identified genes and molecular mechanisms involved in stress responses and longevity. For example, the lifespan extension caused by reduced IIS requires the activity of DAF-16, the FOXO (Forkhead box O) orthologue, which induces entry into larval diapause but also promotes longevity in adults^19^. When IIS is reduced under conditions where dauer-associated processes are inactive in adults, lifespan extension also requires SKN-1, the Nrf1/2/3 orthologue^20^, which increases resistance to various stresses^21^. In addition, reduced IIS causes each of these proteins to accumulate in nuclei, leading to upregulation of target genes involved in longevity, stress responses, metabolism, and the extracellular matrix^20^,^22^,^23^,^24^. In *C. elegans*, SKN-1 is required for lifespan to be extended by a variety of different interventions^20^,^24^,^25^,^26^,^27^,^28^,^29^. Under oxidative stress conditions, PMK-1, a p38 mitogen-activated protein kinase (MAPK), phosphorylates SKN-1, leading to its nuclear accumulation and target gene expression^30^. In addition to the longevity modulating effect of SKN-1, recent studies have also demonstrated its critical roles in protein homeostasis under conditions of reduced translation or proteasome activity^31^,^32^ or increased endoplasmic reticulum (ER) stress^33^. SKN-1 then selectively induces distinct but partly overlapping set of its downstream target genes under these diverse conditions.

In this study, we have investigated how SAC and SAMC affect lifespan and oxidative stress resistance of *C. elegans*. In addition, we examined their effects on pathways regulated by the DAF-16/FOXO and SKN-1/Nrf transcription factors. We also tested whether SAC and SAMC could mimic a dietary restriction (DR)-like environment, which is strongly linked to longevity of various species including *C. elegans*. Finally, we investigated effect of various OSCs from garlic and their analogs on induction of a *gst-4p::GFP* transgene, an indicator of SKN-1 activity.

## Results and Discussion

### SAC and SAMC extend *C. elegans* lifespan under normal conditions

**Figure 1:**
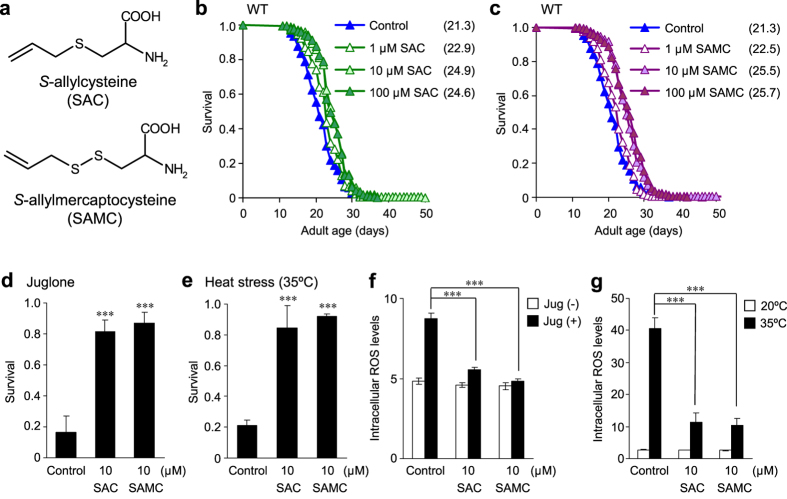
SAC and SAMC increase lifespan and resistance to oxidative- or heat-stress of wild-type *C. elegans*. (**a**) Chemical structures of SAC and SAMC. (**b,c**) Survival curves of wild-type adults treated with SAC (**b**) or SAMC (**c**) at 20 °C. Composites of four replicates are shown respectively, with mean lifespans indicated in parentheses. Statistics are provided in Supplementary Table S1. (**d–g**) Synchronized day-1 wild-type adults were treated with H_2_O (control), SAC or SAMC for 48 hours at 20 °C and then subjected to oxidative stress (250 μM juglone (Jug) for 2 hours at 20 °C) or heat stress (35 °C for 7 hours). (**d,e**) Survivals after each stress treatment were scored after a 16 hours recovery on NGM agar seeded with *E. coli* OP50. Data are represented as mean ± SD from three independent experiments. Total number of animals tested: for the oxidative stress assays (Control, n = 218; SAC, n = 211; SAMC, n = 226) and for the heat stress assays (Control, n = 208; SAC, n = 226; SAMC, n = 220). (**f,g**) Intracellular ROS accumulation in individual animal was measured by using CM-H_2_DCFDA. The mean fluorescence intensity of at least 20 animals for each group with or without stress treatment is shown. Error bars represent SEM. ***P < 0.001 (one-way ANOVA with Tukey’s post hoc test).

We first evaluated whether SAC and SAMC (Fig. 1a) influence the lifespan of wild-type *C. elegans* under normal conditions. To eliminate the possibilities that these compounds could affect growth of *E. coli* OP50, and vice versa live bacteria could metabolize these compounds, we used UV-killed *E. coli* OP50 in the lifespan and the following assays. In our lifespan assays, SAC- and SAMC-treatment were begun on the first day of adulthood with concentrations at 1, 10, and 100 μM at 20 °C. As a result, SAC produced significant increase in the mean lifespan of adult animals (7.5% for 1 μM (*P* < 0.001), 17.0% for 10 μM (*P* < 0.001) and 15.6% for 100 μM (*P* < 0.001), Fig. 1b, Supplementary Table S1). Similarly, SAMC-treatment also significantly increased the mean lifespan (5.8% for 1 μM (*P* < 0.05), 19.7% for 10 μM (*P* < 0.001) and 20.9% for 100 μM (*P* < 0.001), Fig. 1c, Supplementary Table S1). On the other hand, SAC and SAMC did not affect the maximum lifespan of wild-type *C. elegans* (Supplementary Table S1). These results would suggest that these compounds influence the death of younger but not older animals. One possible reason for the lack of effects of these compounds on the maximum lifespan is attributed to decreased stability and/or persistence of effects of these compounds because SAC and SAMC were only added to *C. elegans* on the first day of the lifespan experiments. Therefore, it is possible that additional treatments with fresh SAC and SAMC during middle or late period of the lifespan experiments might affect the maximum lifespan. Given that the significant extension of the mean lifespan of wild-type *C. elegans* was achieved at 10 and 100 μM of each compound, we performed the following experiments at these concentrations.

### SAC and SAMC enhance stress resistance and reduce ROS levels under oxidative- and heat-stress conditions

In *C.elegans*, increased lifespan is sometimes associated with improved survival under conditions of oxidative or heat stress^34^,^35^. To investigate whether SAC and SAMC could enhance resistance to stress, we pretreated wild-type adults with 10 μM of SAC or SAMC for 2 days at 20 °C, followed by exposure to oxidative (juglone, an intracellular ROS generator) or heat stress (35 °C). Both SAC- and SAMC-pretreatment increased survival after juglone exposure (Fig. 1d) and heat stress (Fig. 1e) at significantly higher ratio than untreated control. These results indicate that both compounds exert protective roles against oxidative and heat stress in *C. elegans*. Because both juglone treatment and heat shock cause cellular damage by accumulation of ROS, we next investigated whether SAC and SAMC could lower the intracellular ROS level under stress conditions by using CM-H_2_DCFDA, a fluorescent probe that reacts with ROS. The results showed that pretreatment with SAC or SAMC significantly suppresses oxidative or heat stress-induced accumulation of ROS compared to untreated control (Fig. 1f,g), suggesting that the increase in lifespan and stress resistance by SAC- or SAMC-treatment is associated with reduced ROS levels. Since SAC and SAMC have been shown to act as radical scavengers^14^, the increased lifespan and stress resistance by SAC and SAMC could be at least in part due to the direct antioxidant properties. On the other hand, there is increasing evidence that SAC and SAMC modulate pathways involved in oxidative stress response^8^,^16^,^18^. Therefore, we investigated whether the SAC- and SAMC-mediated increase in stress resistance and longevity in *C. elegans* could be produced by activating pathways particularly associated with oxidative stress responses and longevity.

### SAC and SAMC do not affect DAF-16/FOXO activity

**Figure 2:**
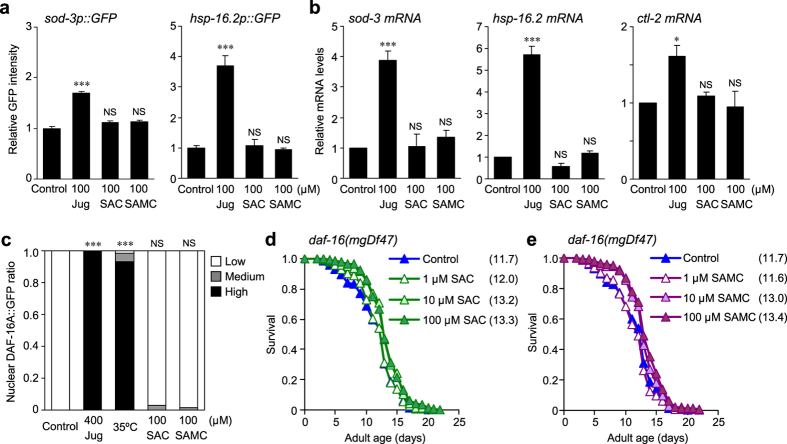
SAC and SAMC do not affect DAF-16 pathway. (**a**) Induction of the *sod-3p::GFP* or *hsp-16.2p::GFP* transgene in animals treated with juglone, SAC or SAMC for 24 hours. GFP intensity in pharynx was quantified by ImageJ. Data are represented as relative fluorescence intensity with SEM (n ≥ 16 for each group). (**b**) Relative mRNA levels of *sod-3* (left), *hsp-16.2* (middle) and *ctl-2* (right) in day-1 wild-type adults treated with juglone, SAC or SAMC for 6 hours (n = 3 of 50 animals) were determined by qRT-PCR. Data are represented as mean ± SEM from three independent experiments normalized to the levels in control. (**c**) Nuclear localization of DAF-16A::GFP in animals treated with H_2_O (control; n = 73), SAC (100 μM; n = 66) or SAMC (100 μM; n = 63) for 24 hour. Juglone (400 μM for 1 hour; n = 63) or heat stress (35 °C for 1 hour; n = 73) were used as positive controls. Nuclear localization of DAF-16A::GFP throughout whole body was classified into High, Medium or Low. ***P < 0.001; NS: not significant (chi^2^ test). (**d,e**) Survival curves of the *daf-16(mgDf47)* mutant treated with SAC (**d**) or SAMC (**e**) at 20 °C. Composites of three replicates are shown respectively, with mean lifespans indicated in parentheses. Statistics are provided in Supplementary Table S2. *P < 0.05; ***P < 0.001; NS: not significant (one-way ANOVA with Tukey’s post hoc test).

In *C. elegans*, the evolutionarily conserved DAF-16/FOXO transcription factor regulates many biological processes including stress resistance and longevity^22^,^23^. Therefore, we examined whether SAC and SAMC could have any effect on DAF-16 signaling. We first monitored expression of transgenes in which promoter for DAF-16 target genes *sod-3* (superoxide dismutase) or *hsp-16.2* (small heat shock protein) is fused to green fluorescent protein (GFP), respectively. As shown in Fig. 2a, juglone (positive control) upregulated the expression of both *sod-3p::GFP* and *hsp-16.2p::GFP* transcriptional reporters, whereas no induction of these reporters was observed by SAC- and SAMC-treatment (100 μM each for 24 hours). We also examined expression of endogenous *sod-3*, *hsp-16.2*, and *ctl-2* (catalase) mRNAs by quantitative RT-PCR (qRT-PCR), and found that neither of these genes was activated by these compounds (100 μM each for 24 hours) (Fig. 2b).

To further investigate the effect of SAC and SAMC on DAF-16 signaling, we examined whether SAC and SAMC could promote accumulation of a DAF-16A::GFP translational fusion protein in the nucleus. Like other transcription factors, nuclear localization of DAF-16 is associated with its transcription-activating activity. Exposure to juglone and heat stress resulted in remarkable nuclear localization of DAF-16A::GFP, whereas no nuclear localization of DAF-16A::GFP was observed in animals treated with SAC or SAMC (100 μM each for 24 hours) (Fig. 2c). To further elucidate the involvement of DAF-16 signaling in the effects of SAC and SAMC on nematodes, we performed the lifespan assays using the *daf-16(mgDf47)* mutant. We found that treatments with SAC and SAMC at 10 and 100 μM appeared to prolong survival of the *daf-16(mgDf47)* mutant in early stage of adult life (Fig. 2d,e, Supplementary Table S2), and when we combined three independent assays, significant extension of the mean lifespan was observed in treatments with 10 and 100 μM of these compounds, although this lifespan extension was reduced compared to wild-type (Supplementary Table S2). In addition, SAC and SAMC did not extend the maximum lifespan of this mutant presumably due to the same reason as observed in wild-type (Supplementary Table S2). Taken together, our results show that SAC- and SAMC-mediated increase in lifespan and stress resistance appears to be in part independent of DAF-16 signaling.

### SAC and SAMC promote longevity by modulating SKN-1

**Figure 3:**
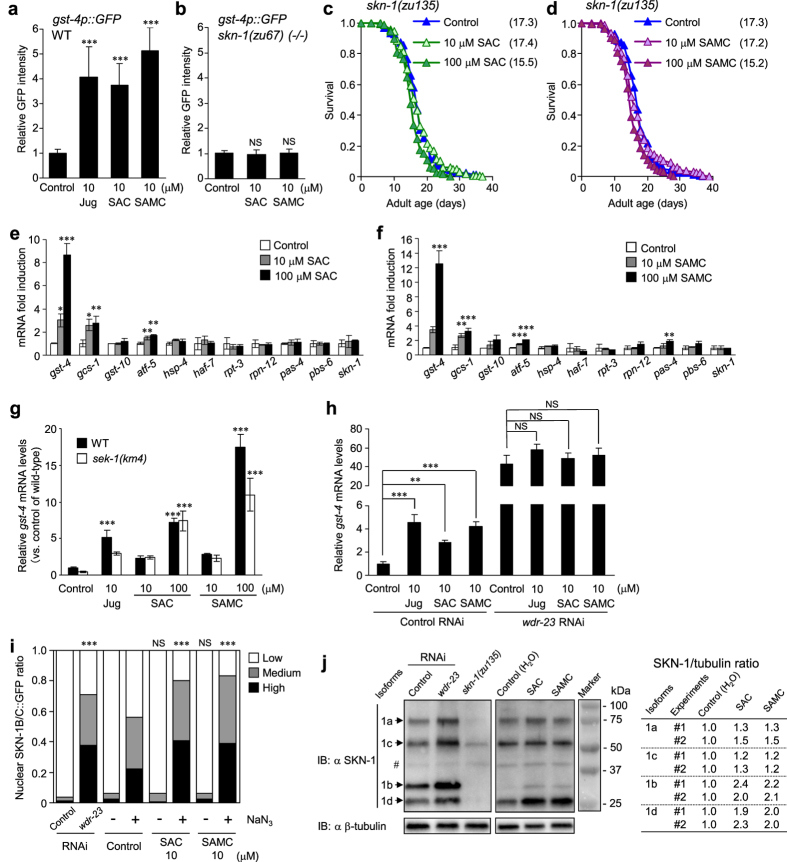
SAC and SAMC modulate SKN-1 pathway. (**a**,**b**) Induction of *gst-4p::GFP* transgene in day-1 adults of the wild-type background (**a**) or the *skn-1(zu67)* mutant (**b**) treated with juglone, SAC or SAMC (24 h). Data represent relative fluorescence intensity throughout whole body with SD (n ≥ 20). (**c,d**) Lifespan of the *skn-1(zu135)* mutant treated with SAC (**c**) or SAMC (**d**) at 20 °C. Composites of three replicates with mean lifespans in parentheses. Statistics are provided in Supplementary Table S3. (**e,f**) Relative mRNA levels of the indicated SKN-1 targets in day-1 wild-type adults treated with SAC (**e**) or SAMC (**f**) (24 h). (**g**) Relative *gst-4* mRNA levels in day-1 adults of the *sek-1(km4)* mutant treated with juglone, SAC or SAMC (24 h). (**h**) Effect of *wdr-23* RNAi on endogenous *gst-4* mRNA levels in day-1 wild-type adults treated with juglone, SAC or SAMC (24 h). (**e–h**) Data represent mean ± SD (n = 3 of 50 animals). (**i**) Nuclear localization of SKN-1B/C::GFP in L4 animals pretreated with SAC or SAMC from the L4 stage of parental generation, followed by treatment with or without NaN_3_. *wdr-23* RNAi was used as a positive control. SKN-1B/C::GFP in intestinal nuclei was classified into High, Medium or Low. ***P < 0.001 (for *wdr-23* RNAi, n = 106 vs. Control RNAi, n = 102, for the NaN_3_ treatment, SAC, n = 142; SAMC, n = 166 vs. Control, n = 151), NS: not significant (without NaN_3_, Control, n = 130; SAC, n = 131; SAMC, n = 135) (chi^2^ test). (**j**) Immunoblotting of endogenous SKN-1. (Left) Whole lysates (4.6 μg/lane) from 300 day-1 adults of the *rrf-3(pk1426)* mutant treated with control or *wdr-23* RNAi, or of the *skn-1(zu135)* homozygous mutant. (Middle) Whole lysates (15.0 μg/lane) from 1,000 L4 wild-type treated with SAC or SAMC from the L4 stage of parental generation. The blots detected with antibodies against SKN-1 (top) or β-tubulin (bottom). Predicted SKN-1 isoforms (1a–1d) are indicated according to their estimated molecular weights reported in WormBase. (Right) Relative band intensity against β-tubulin of two experiments normalized to the levels in control of each isoform. The blot are data of experiment-1. #: Non-specific band. *α*: antibody against. *P < 0.05; **P < 0.01; ***P < 0.001; NS: not significant (one-way ANOVA with Tukey’s post hoc test).

In *C. elegans*, the transcription factor SKN-1/Nrf plays a critical role in promoting oxidative stress resistance and longevity by upregulating numerous genes, including phase II detoxification enzymes^24^,^26^,^30^,^36^,^37^. To investigate whether SAC- and SAMC-treatment could modulate SKN-1 activity, we first examined the effect of SAC and SAMC on expression of *gst-4* (glutathione *S*-transferase) gene, one of the key phase II enzyme genes that is strongly activated in response to oxidative stress^36^,^37^. We treated the transgenic animals, which contains a *gst-4p::GFP* transcriptional reporter transgene, with juglone (positive control), SAC and SAMC. All these treatments resulted in a dramatic increase in GFP expression compared to untreated control (Fig. 3a). To confirm whether the *gst-4p::GFP* induction by SAC- and SAMC-treatment could require SKN-1, we treated the *skn-1*(*zu67*) mutant, which carries the *gst-4p::GFP* transgene, with SAC and SAMC. We found that no induction of *gst-4p::GFP* was observed by these compounds in this mutant (Fig. 3b), indicating that the induction of *gst-4p::GFP* by SAC and SAMC is completely dependent upon SKN-1.

It has been demonstrated that *skn-1* loss-of-function mutants have shortened lifespans, and in contrast, that increased expression or activity of SKN-1 increases *C. elegans* lifespan^24^. We next examined whether SAC- and SAMC-mediated extension of lifespan requires SKN-1. We treated the *skn-1*(*zu135*) mutant with SAC and SAMC, and found that both compounds failed to increase the mean lifespan of this mutant compared to untreated control (Fig. 3c,d, Supplementary Table S3). Instead, this mutation shortened the mean lifespan in the presence of 100 μM SAC or SAMC (Fig. 3c,d, Supplementary Table S3). This may indicate that SAC and SAMC have caused toxicity to the *skn-1(zu135)* mutant. Even in wild-type *C. elegans*, SAC and SAMC might partly act as mild stressors. On the other hand, the toxic effects of these compounds might be offset by activation of SKN-1, leading to induction of stress defense genes such as *gst-4* and eventually extension of mean lifespan. Together, these results suggest that *skn-1* is required for the SAC- and SAMC-mediated lifespan extension.

In *C. elegans*, SKN-1 is activated in response to diverse interventions, such as oxidative- and ER-stress, and reduced translation and proteasome activity, leading to partially overlapping but distinct set of target gene expression^26^,^27^,^31^,^32^,^33^. To investigate how SAC- and SAMC-treatment could affect expression of SKN-1 target genes, we examined mRNA levels of some SKN-1 targets related to response against oxidative- or ER-stress, and reduced translation elongation or proteasome activity. SAC- and SAMC-treatment on wild-type animals significantly induced some oxidative stress defense genes, *gst-4* and *gcs-1* (γ-glutamylcysteine synthase heavy chain^26^,^30^), except *gst-10*^27^ (Fig. 3e,f). Additionally, the *skn-1*-dependent ER and oxidative stress-related transcription factor *atf-5* (a mammalian bZIP transcription factors ATF4^27^,^33^) was also induced by these compounds (Fig. 3e,f). On the other hand, SAC and SAMC did not increase transcription of *hsp-4* (heat shock protein) and *haf-7* (an ortholog of human ATP-binding cassette B9, ABCB9) (Fig. 3e,f), which are induced by SKN-1 in response to ER stress and reduced translation, respectively^27^,^31^,^32^,^33^.

Knockdown of some proteasome subunit genes by RNAi induces *skn-1*-dependent expression of endogenous *gst-4* and *gst-10*^32^. Additionally, the amyloid-binding dye Thioflavin T (ThT) has been shown to extend *C. elegans* lifespan dependent upon *skn-1* and also *hsf-1* (heat shock factor 1), which promotes protein homeostasis^38^. ThT also suppresses aggregation of Amyloid-β (3-42) peptide and polyglutamine, which are associated with Alzheimer’s disease and several neurological conditions, respectively, in *C. elegans* models^38^. One possibility is that undesirable accumulation of aggregated or misfolded proteins in cells might activate SKN-1 to induce its targets associated with protein homeostasis. In contrast, our data indicated that SAC and SAMC did not substantially affect mRNA levels of various components of the proteasomal complex; *rpt-3* (an ATPase subunit of the 19S proteasome), *rpn-12* (a non-ATPase subunit of the 19S proteasome), *pas-4* (an alpha-rings subunit of the 20S proteasome), and *pbs-6* (a beta-rings subunit of the 20S proteasome)^32^ (Fig. 3e,f). We further examined the effect of SAC and SAMC on the 26S proteasome activity and found that these compounds had no effect on its activity (Supplementary Fig. S1), suggesting that these compounds appear to activate SKN-1 through a mechanism uncoupled from protein homeostasis. Taken together, these results suggest that SAC and SAMC may act primarily on oxidative stress response genes regulated by SKN-1, and that this may confer the increased lifespan and stress resistance associated with SAC and SAMC treatment.

We also tested the possibility of whether SAC and SAMC could induce expression of *skn-1* itself, thus leading to induction of its target genes. Results showed that these compounds had no effect on *skn-1* mRNA expression (Fig. 3e,f). Therefore, we next examined whether SAC and SAMC could modulate SKN-1 activity at the protein level. Under oxidative stress conditions, SKN-1 is activated by p38 MAPK pathway signaling^30^. p38 MAPK directly phosphorylates specific sites within SKN-1, which then accumulates in the nucleus and activates oxidative stress defense genes such as *gcs-1*^26^,^30^. Downstream of or in parallel to this regulation, WDR-23 (WD40 repeat protein) physically interacts with SKN-1 and CUL-4/DDB-1 ubiquitin ligase complex in the nucleus, which presumably ubiquitinylates SKN-1 protein and targets it for proteasomal degradation^37^. To elucidate how SAC and SAMC modulate SKN-1 activity, we first examined the effect of these compounds on the p38 MAPK pathway. We treated the *sek-1(km4)* mutant, a gene encoding p38 MAPKK that function is essential for the p38 MAPK pathway, with SAC and SAMC and examined the effect of these compounds on *gst-4* mRNA expression. As a result, these compounds also activated transcription of *gst-4* in this mutant as well as that of wild-type (Fig. 3g), suggesting that the SAC- and SAMC-mediated activation of *gst-4* transcription, which requires *skn-1* (Fig. 3b), might be independent of the p38 MAPK pathway. This was surprising because induction of *gcs-1* is drastically inhibited in the *sek-1* (p38 MAPKK) and *pmk-1* (p38 MAPK) mutants^30^. On the other hand, it is also indicated that transcription of *gcs-1* is activated in the *sek-1(km4)* mutant when several genes (e.g. *C48B6.2*, *phi-43* or *wdr-23*) are knocked down by RNAi^31^. In addition, another study also demonstrated that *wdr-23* RNAi robustly induced *gst-4* transcription in the *sek-1(km4)* mutant^37^.

Therefore, we next assessed the possibility that SAC and SAMC could activate SKN-1 and its target expressions through regulation by WDR-23. To test this idea, we examined the effect of *wdr-23* knockdown by RNAi on the SAC- and SAMC-induced *gst-4* mRNA expression. As shown in Fig. 3h, *wdr-23* RNAi drastically caused *gst-4* mRNA expression in untreated control animals compared with that of control RNAi, and no additional increase in *gst-4* expression was observed in the SAC- or SAMC-treated animals. This suggests that SAC and SAMC might modulate SKN-1 activity by regulating WDR-23 or its interaction with SKN-1, or possibly by stabilizing SKN-1.

Loss of WDR-23 function causes nuclear accumulation of SKN-1 in intestine, and increases SKN-1 protein levels, leading to activation of target genes^37^. Therefore, we next assessed the possibility whether SAC- and SAMC-treatment could promote nuclear accumulation of SKN-1. We examined the effect of these compounds on subcellular distribution of a SKN-1B/C::GFP translational fusion protein that encodes two of three SKN-1 isoforms. We treated L4 animals with SAC or SAMC, and then measured nuclear accumulation of SKN-1B/C::GFP at L3 or L4 stages of the next generation. Results showed that SAC- and SAMC-treatment did not detectably increase nuclear accumulation of SKN-1B/C::GFP under normal conditions (Fig. 3i), suggesting that these compounds do not substantially affect nuclear localization of SKN-1. On the other hand, it is also possible that hypochlorite treatment for the preparation of L1 animals of the next generation may affect the inducibility of nuclear SKN-1 or levels of SKN-1 protein in later larval stages, leading to a failure of detection of SKN-1B/C::GFP nuclear localization. To address this possibility, we treated L4 animals of the next generation with acute oxidative stress, 2% NaN_3_ (as a positive control of SKN-1B/C::GFP nuclear localization) for 15 min after pretreatments with SAC or SAMC. Results showed that this acute oxidative stress caused drastic nuclear accumulation of SKN-1B/C::GFP as indicated in ref. 36, and population of animals with nuclear SKN-1B/C::GFP slightly but reproducibly increased after exposure to 2% NaN_3_ when they were pretreated with SAC or SAMC (Fig. 3i). Taken together, these results implicate that SAC and SAMC do not cause nuclear accumulation of SKN-1 directly under normal conditions, but may facilitate nuclear accumulation of SKN-1 in response to acute oxidative stress by possibly defending it against degradation through WDR-23 regulation.

Consistent with our observation, some studies demonstrated that reduced mTORC1 (mammalian target of rapamacin complex) and tunicamicin-induced ER stress also upregulated SKN-1 targets without robust accumulation of this transcription factor in the nucleus^28^,^33^. In addition, tunicamicin treatment also causes increase of intracellular abundance of SKN-1 protein^33^. Therefore, we investigated whether SAC- and SAMC-treatment could increase intracellular SKN-1 protein levels. To test this idea, total amount of SKN-1 protein in SAC- or SAMC-treated animals was assessed by western blotting using a polyclonal SKN-1 antibody, which was raised against SKN-1c isoform and should detect all of main SKN-1 isoforms (SKN-1a, 1b and 1c). This antibody recognized multiple bands, and four of these increased by *wdr-23* RNAi and decreased in the *skn-1(zu135)* mutant, suggesting that these four bands might correspond to each SKN-1 isoform (1a~1d) (Fig. 3j left). As shown in Fig. 3j (middle and right), SAC and SAMC slightly but reproducibly increased protein levels of species that may correspond to the SKN-1b (2.0~2.4-fold) and SKN-1d (1.9~2.3-fold) isoforms, respectively. SKN-1b is principally expressed in the ASI neurons, which sense food availability and influence metabolism, and are involved in dietary restriction-induced longevity^26^,^39^. Even though the transcript of *skn-1d* has been reported in WormBase, neither expression nor function of the smallest isoform has been described. At the moment, the underlying mechanisms of the selective increase in these two SKN-1 isoforms by SAC and SAMC are still unclear.

In mammals, Kelch-like ECH-associated protein 1 (Keap1) directly binds to Nrf2, and targets it for polyubiquitination and then proteasomal degradation^21^. Some electrophilic compounds including sulforaphane have been demonstrated to bind to some cysteines in Keap1, and thus leading to release of active Nrf2^40^. *C. elegans* lacks a Keap1 ortholog^41^, and instead WDR-23 directly interacts with SKN-1 and targets it for proteasomal degradation^37^. Currently, we have been investigating the possibility whether these compounds could bind to redox-reactive cysteines in WDR-23 or SKN-1 itself.

### SAC and SAMC do not affect body size and reproduction, but enhance food intake of wild-type *C. elegans*

**Figure 4:**
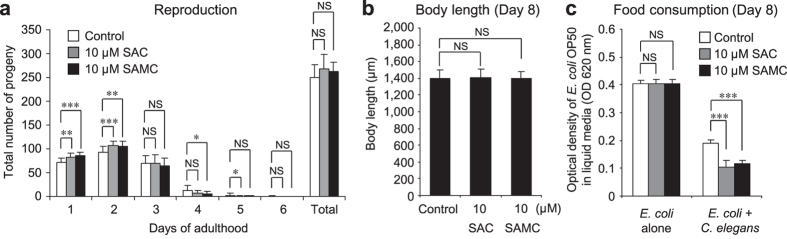
SAC and SAMC do not affect body size and reproduction, but enhance food intake of wild-type *C. elegans*. (**a**) For the reproduction assays, wild-type L4 animals were treated with H_2_O (control; n = 17), SAC (n = 19) or SAMC (n = 22) until reproduction period was ceased. Data represent the mean value of daily or total number of progeny from individual animals with SD. (**b**) The body length of animals treated with H_2_O (control; n = 85), SAC (n = 87) or SAMC (n = 91) for 8 days was measured by ImageJ. Data represent mean ± SD. (**c**) For the food consumption assays, after 8 days of treatment with H_2_O (control), SAC, or SAMC, OD 620 nm of liquid medium containing total 50 animals was measured with a spectrophotometer. Data represent mean ± SD (n = 4 of 50 animals). *P < 0.05; **P < 0.01: ***P < 0.001; NS: not significant (one-way ANOVA with Tukey’s post hoc test).

It has been revealed that reducing food intake (dietary restriction, DR) extends lifespan of a wide range of species, including *C. elegans*^39^,^42^,^43^,^44^,^45^. In *C. elegans*, DR-induced extension of lifespan appears to require *skn-1b* particularly in the ASI neurons^26^,^39^. Our findings also suggest that SAC and SAMC increased levels of the SKN-1b isoform (Fig. 3j). Therefore, we considered the possibility that SAC and SAMC might activate SKN-1 in the ASI neurons to produce DR-like state, leading to lifespan extension. Since diet-restricted animals also exhibit reduced brood size, extended reproductive period, and smaller body size^39^, we examined the influence of SAC- and SAMC-treatments on reproductive capacity. The results showed that the animals treated with SAC or SAMC for 8 days exhibited a significant increase in progeny production on the 1^st^ and 2^nd^ day of reproductive period, although the total number of progeny was not statistically significant compared to untreated animals (Fig. 4a). Furthermore, neither SAC nor SAMC affected the reproductive period of *C. elegans* (Fig. 4a), suggesting that SAC and SAMC do not affect the reproductive capacity of *C.elegans*. We next examined whether SAC and SAMC could affect *C. elegans* body size. The result showed that wild-type animals raised in the presence of either SAC or SAMC for 8 days did not exhibited any differences in body length compared to untreated animals (Fig. 4b), suggesting that SAC and SAMC also seem to be unrelated with DR with respect to body length.

To assess whether SAC- or SAMC-treatment could cause reduced food intake, we examined the level of food (UV-killed *E. coli* OP50) consumption by measuring the optical density (OD) of wells containing equal numbers of animals (n = 50) after 8 days of treatments with SAC or SAMC. The mean values of OD 620 nm of wells without *C. elegans* were comparable among treatments, suggesting that these compounds do not affect food concentration directly (Fig. 4c). On the other hand, SAC- and SAMC-treated animals showed a significant increase in food consumption compared to untreated control (Fig. 4c). This phenomenon became visually apparent after about 5 days of treatment (unpublished data). One likely explanation for this phenomenon is that SAC- and SAMC-mediated activation of SKN-1 could slow aging of *C. elegans*, and this health promoting effects of these compounds may lead to the elevated food consumption of this organism, despite enhanced food consumption itself produces more ROS in cells in general. Taken all together, these results indicate that, at least for parameters investigated here, SAC and SAMC do not extend *C. elegans* lifespan by producing a DR-like state.

### The thioallyl structure and disulfide bond in garlic-derived OSCs are important for SKN-1 activation

Including SAC and SAMC, numerous OSCs, such as *S*-alk(en)ylcysteines, *S*-alk(en)ylcysteine sulfoxides, γ-glutamyl-*S*-alk(en)ylcysteines, and allylsulfides, have been identified from garlic^46^. Some of those have been shown to have diverse pharmacological properties as SAC and SAMC, such as radical scavenging activity, chemopreventive activity, hepatoprotective activity, neurotropic activity, and lipid reducing activity^8^,^10^,^11^,^47^,^48^. Moreover, a recent study reported that diallyl trisulfide (DATS), one of the allylsulfides derived from garlic, is also able to induce *gst-4* gene expression under control of *skn-1* with extended longevity in *C. elegans*^49^. Therefore, we considered the question of whether other OSCs in garlic might activate SKN-1/Nrf as well as SAC and SAMC, and whether their structures might be correlated with this activity. To address this possibility, we tested the effect of garlic-derived OSCs and their analogs on induction of *gst-4p::GFP* transgene. As shown in Table 1, of 23 compounds tested, 5 compounds (SAC; 4.9-fold (*p* < 0.001), SAMC; 8.1-fold (*p* < 0.001), DADS (diallyldisulfide); 3.4-fold (*p* < 0.001), DATS; 9.1-fold (*p* < 0.001), GSAMC (γ-glutamyl-*S*-allylmercaptocysteine); 3.0-fold (*p* < 0.001)) produced a significant increase in *gst-4p::GFP* expression compared to untreated control. Importantly, all these compounds commonly have the thioallyl structure. Moreover, there was a positive correlation between the number of disulfide bonds and *gst-4p::GFP* induction levels as in the case of SAC < SAMC, and DAS < DADS < DATS.

Among compounds containing the allyl structure, alliin (*S*-allylcysteine sulfoxide), in which the sulfur atom of SAC forms sulfoxide group, and OAS (*O*-allylserine), in which the sulfur atom of SAC is substituted by oxygen, exhibited no *gst-4p::GFP* induction. In addition, GSAC (γ-glutamyl-*S*-allylcysteine) and GSAMC, in which glutamic acid is attached to *α*-amino group of cysteine, exhibited weaker *gst-4p::GFP* inducible activity than SAC and SAMC, respectively. Similarly, SAHC (*S*-allylhomocysteine) and SAMHC (*S*-allylmercaptohomocysteine) had no significant effect on the activity.

Taken together, we found out the following structurally important factors that affect *gst-4p::GFP* inducible activity; i) the thioallyl structure is essential; ii) an increasing number of sulfur atoms in sulfide bonds leads to enhanced activity; iii) the sulfur atom adjacent to the allyl group and iv) cysteine structure are also important factors influencing the activity. Given that SAC and SAMC possibly stabilize SKN-1 by suppressing the interaction between SKN-1 and WDR-23 through binding to reactive cysteines in either of these proteins, it would be interesting to see whether these activity-related factors are closely linked to this event.

The finding that the increasing number of disulfide bonds correlates with the *gst-4p::GFP* inducible activity raises the question of whether the number of disulfide bonds in these compounds might also coorelate with their protective effect. To address this, we performed oxidative stress assays using SAC, SAMC, DADS and DATS (10 μM each), and found significantly higher survivals after treatment with DADS (41.9 ± 3.1%; *P* < 0.001) and DATS (41.4 ± 2.6%; *P* < 0.001) compared to DMSO control (16.0 ± 3.4%) (Table 1, Supplementary Fig. S2). However, neither DADS nor DATS treatment increased survival as robustly as SAC (83.2 ± 6.2%; *P* < 0.001 vs. DADS and DATS) or SAMC (90.2 ± 4.0%; *P* < 0.001 vs. DADS and DATS) (Table 1, Supplementary Fig. S2), indicating that there is no positive correlation between the number of disulfide bonds and stress resistance capacity. Similar results were obtained in the heat stress assays (Table 1, Supplementary Fig. S3). Because DATS treatment at higher concentration (100 μM) caused death of adult animals within 24 hours, this toxicity by DATS might lead to the lower survivals in the oxidative stress assay in spite of its highest *gst-4p::GFP* inducible activity. Alternatively, it is also possible that there is an optimal level of SKN-1 activation which, if exceeded may be deleterious. On the other hand, treatment with SAC and SAMC at 100 μM still caused the increased mean lifespan (Fig. 1b,c) and higher survivals (88.2 ± 10.4% for SAC, 91.1 ± 9.3% for SAMC (N = 3 experiments using more than 50 animals each)) in the oxidative stress assays. These results implicate that SAC and SAMC can be treated at higher concentrations with less toxicity, thus leading to a superior protective effect compared to DATS.

Interestingly, consistent with our findings, some previous studies using garlic-derived OSCs also reported the importance of the thioallyl structure and/or the number of sulfur atoms in sulfide bonds on diverse biological activities. For example, the study investigating chemopreventive activity of *S*-alk(en)ylcysteines and these disulfide derivatives indicated that thioallyl compounds, including SAC, were the most effective for colon cancer prevention^50^. Another study investigating neurotropic activity of *S*-alk(en)ylcysteines, *S*-alk(en)ylcysteine sulfoxides, γ-glutamyl-*S*-alk(en)ylcysteine, and their analogs also indicated that only thioallyl compounds, such as SAC, SAMC, DAS, DADS, alliin, and GSAC, were effective on the survival of cultured rat hippocampal neurons^10^. The study of radical scavenging capacity of some OSCs also revealed that thioallyl structure and the number of the sulfur atoms contribute to the activity^47^. Although direct target(s) of these thioallyl compounds and their underlying mechanisms are still unclear, the notable consistency of observations derived from these and our studies suggests that the thioallyl compounds from garlic play important roles in diverse biological processes including the SKN-1/Nrf pathway.

In conclusion, we have reported that SAC and SAMC increase resistance to oxidative stress and longevity of the nematode *C. elegans*. These beneficial effects of SAC and SAMC are most likely conferred by modulation of SKN-1/Nrf activity and selective activation of its downstream targets involved in oxidative stress defense. Taken together our findings suggest that at least a portion of the multiple health promoting activities of garlic and its constituents, especially those from thioallyl compounds, could be explained by SKN-1/Nrf activation. Furthermore, our study may provide the possibility of applications of natural thioallyl compounds to the development of nutraceutical products and drugs targeting Nrf pathway.

## Methods

### Reagents

SAC and SAMC were synthesized as in refs. 51 and 7, respectively, stored in water solution and added to culture medium at various concentrations.

### Strains and culture of *C. elegans*

Nematode strains used in this study are listed in Supplementary Table S4. Each strain was maintained at 20 °C on nematode growth medium (NGM) agar plates carrying a lawn of *E. coli* OP50 (*Caenorhabditis* Genetics Center) according to ref. 52. Unless otherwise stated, animals for each assay were raised according to the following procedure. Briefly, to synchronize growth of *C. elegans*, gravid hermaphrodites were treated with sodium hypochlorite and resulting eggs were kept overnight at 20 °C for hatching in S-complete liquid medium. Synchronized L1 animals were then transferred to a 96-well plate in S-complete liquid medium containing amphotericin B (0.1 μg/mL) and the UV-killed *E. coli* OP50 (1.2 × 10^9^ bacteria/mL), sealed to prevent evaporation, and kept at 20 °C^53^. UV killing of *E. coli* OP50 was done using a stratalinker (9999 J/m^2^, Stratagene, La Jolla, CA) to exclude any effects of the test compounds on bacterial growth, and unexpected metabolism of these compounds by live bacteria^54^. 5-fluoro-2′-deoxyuridine (FUdR, 0.12 mM) was added 42–45 hours after seeding to prevent self-fertilization. Thirty micro liters of SAC or SAMC solution, or H_2_O as solvent control were added on the first day of adulthood at final concentrations ranging from 1 to 100 μM, respectively.

### Lifespan assays

All lifespan assays were started on the first day of adulthood and performed at 20 °C. To avoid starvation, an adequate amount of the UV-killed OP50 was added to each well during assays. Counting of surviving or dead animals was performed daily using a microscope on the basis of movement until all animals had died. Before counting each plate was shaken for one minute on a plate shaker to facilitate observation of movement.

### Stress resistance assays

Synchronized day-1 wild-type adults were pretreated with H_2_O, SAC or SAMC (10 μM each) for 48 hours at 20 °C. For the oxidative stress assays, the animals were washed with phosphate-buffered saline with 1% Tween 20 (PBST) three times before treating with a ROS generator, juglone (250 μM, Sigma-Aldrich, St. Louis, MO), for 2 hours at 20 °C. For the thermo-tolerance assays, the animals were incubated at 35 °C for 7 hours, and then washed with PBST three times. After a 16 hours recovery period on NGM agar, the survival was determined by touch-provoked movement. Animals were scored as dead when they failed to respond to touching with a platinum wire pick.

### Measurement of intracellular ROS in *C. elegans*

To measure intracellular ROS accumulation level in animals after both the oxidative- and the heat-stress treatment, the surviving animals were incubated in the presence of 5-(and-6)-chloromethyl-2′,7′-dichlorodihydrofluorescein diacetate acetyl ester (CM-H_2_DCFDA, 50 μM, Invitrogen, Carlsbad, CA) in PBST for 1 hour at 20 °C. CM-H_2_DCFDA is a cell permeable substance which is intracellularly converted to H_2_DCFs. This nonfluorescent probe can be oxidized by interaction with intracellular ROS to yield the fluorescent dye DCF. After washing with PBST, the animals were mounted onto microscope slides coated with 2% agarose, anesthetized with tetramisole (5 mM), and capped with cover slides. Fluorescence images were collected with a BIOREVO BZ-9000 fluorescent microscope (KEYENCE, Osaka, Japan) using the GFP-BP filter set with excitation at 470 nm and emission at 535 nm. The fluorescence intensity of whole body was quantified as mean pixel density by using ImageJ software (NIH, Bethesda, MD).

### Transgenic reporter assays

Synchronized day-1 adults of the transgenic strains carrying an inducible green fluorescence protein (GFP) reporter transgene for *sod-3* (CF1553), *hsp-16.2* (CL2070) or *gst-4* (CL2166 or CL691(*skn-1(zu67)*)) were treated with H_2_O, SAC or SAMC (10 or 100 μM each) for 24 hours at 20 °C. Juglone (10 or 100 μM) was used as positive control. GFP fluorescence images were collected with randomly selected animals as described in the measurement of intracellular ROS. For the *sod-3p::GFP* and *hsp-16.2p::GFP* reporters, GFP fluorescence from pharynx was quantified by ImageJ. For the *gst-4p::GFP* reporter, GFP fluorescence from whole body was quantified.

### Quantitative real-time reverse transcription PCR (qRT-PCR)

Synchronized day-1 adults of wild-type or KU4 *(sek-1(km4))* strains were treated with H_2_O, SAC or SAMC (10 or 100 μM each) for 6 or 24 hours at 20 °C. Total RNA was extracted from about 50 animals with TRIzol (Invitrogen). Complementary DNA was produced using random 6-mer and oligo (dT) primer. qPT-PCR was performed using SYBR green as the detection method. Expression levels of each mRNA relative to *act-1* gene were calculated with the comparative 2^−ΔΔCT^ method. Primer sequences used in this study are listed in Supplementary Table S5.

### Feeding RNAi

RNAi was performed in a 96-well plate format by feeding *E. coli* HT115 expressing RNAi for either *wdr-23* (clone ID: CUUkp3300D063Q, Source BioScience, Nottingham, UK) or control (pL4440) to nematodes. Synchronized L1 animals were raised in S-complete liquid medium containing amphotericin B (0.1 μg/mL), ampicillin (100 μg/ml), isopropyl *β*-D-1-thiogalactopyranoside (IPTG, 1 mM) and 1.2 × 10^9^ bacteria/mL of an overnight culture of RNAi bacteria induced by IPTG for 1 hour. The animals were grown at 20 °C throughout the assay. FUdR (0.12 mM) was added 42–45 hours after seeding. On the first day of adulthood, the animals were treated with H_2_O, SAC or SAMC (10 μM each) for 24 hours at 20 °C, and expression levels of *gst-4* mRNA were determined by qRT-PCR.

### Nuclear localization DAF-16 or SKN-1

Synchronized day-1 adults of the strains LD1482 or LD001 carrying a transgene that expresses DAF-16A::GFP or SKN-1B/C::GFP fusion protein, respectively, were treated with H_2_O, SAC or SAMC (10 or 100 μM each) at 20 °C. For the DAF-16A::GFP reporter, each treatment was performed for 24 hours. For the SKN-1B/C::GFP reporter, synchronized L4 animals were treated with H_2_O, SAC or SAMC (10 μM each) for 16 hours at 20 °C. The following day, eggs were harvested by hypochlorite treatment, and progeny were further treated with same compound as each parent and allowed to develop to the L4 stage. After washing with PBST the animals were additionally challenged without or with 2% NaN_3_ for 10 min. As a control experiment, synchronized L1 animals of LD001 strain were treated with either control or *wdr-23* RNAi as described above, and then analyzed on the first day of adulthood.

Subcellular distributions of DAF-16A::GFP or SKN-1B/C::GFP were microscopically-classified into “Low”, no visible nuclear localization, “Medium”, nuclear localization visible only in anterior and/or posterior of body, or “High”, strong nuclear localization visible throughout the body or intestine, respectively.

### Western blot analysis

Synchronized wild-type L4 animals were treated with H_2_O, SAC or SAMC (10 μM each) for 16 hours at 20 °C. The following day, eggs were harvested by hypochlorite treatment, and progeny were further treated with same compound as parent and allowed to develop to the L4 stage. The animals (~1,000 animals per condition) were sonicated in 10 volumes of buffer (50 mM Tris-HCl, pH7.6, 50 mM NaCl, 1% sodium dodecyl sulfate and 1× Halt protease and phosphatase inhibitor cocktail (Thermo scientific)) with a Bioruptor UCW310 (BM Equipment, Tokyo, Japan). Homogenates of total protein were harvested after centrifugation at 16,100 × g for 5 min. Protein concentrations were determined with a XL-Bradford kit (APRO science, Tokushima, Japan) after diluted in SDS-PAGE sample buffer. Fifteen μg of protein samples were applied and separated by SDS-PAGE, and detected by immunoblotting with a polyclonal antibody against SKN-1 (1:2000; JDC7^33^) and *β*-tubulin (1:1000; 014-25041; Wako). As control experiments, whole lysates from the *rrf-3(pk1426)* mutant treated with either control or *wdr-23* RNAi from L1 state or the *skn-1(zu135)* mutant were prepared on day-1 adulthood and analyzed. Blots were visualized with a ChemiDoc MP (BioRad, Hercules, CA) and densitometrical analysis was performed using Image Lab software (BioRad).

### Reproduction assays

Synchronized wild-type L4 animals were individually transferred to wells containing H_2_O, SAC or SAMC (10 μM each), and allow laying eggs for 24 hour at 20 °C. The adult animals were transferred to new wells daily until reproduction period was ceased. The number of progeny from individual animal was counted when they raised to the L2 or L3 stage.

### Body length and food consumption assays

Synchronized wild-type day-1 adults were treated with H_2_O, SAC or SAMC (10 μM each) for 8 days at 20 °C. For the body length assays, the animals were collected, and photographs were taken. The body length of individual animal was analyzed using ImageJ. For the food consumption assays, the liquid medium containing total 50 animals was collected and values of optical density at 620 nm were measured with a multiskan spectrophotometer (Labsystems, Helsinki, Finland).

### Statistical analysis

Statistical analysis was performed using KyPlot 5.0 software (KyPlot, Tokyo, Japan). For the lifespan assays, *P*-values were determined by log-rank test. For the nuclear localization of DAF-16A::GFP or SKN-1B/C::GFP, a chi^2^ test was used. One-way analysis of variance (ANOVA) with Tukey’s post hoc analysis was used for other assays. Differences were considered significant at *P* < 0.05.

## Additional Information

**How to cite this article**: Ogawa, T. *et al.* Natural thioallyl compounds increase oxidative stress resistance and lifespan in *Caenorhabditis elegans* by modulating SKN-1/Nrf. *Sci. Rep.* **6**, 21611; doi: 10.1038/srep21611 (2016).

## Supplementary Material

Supplementary Information

## Figures and Tables

**Table 1:**
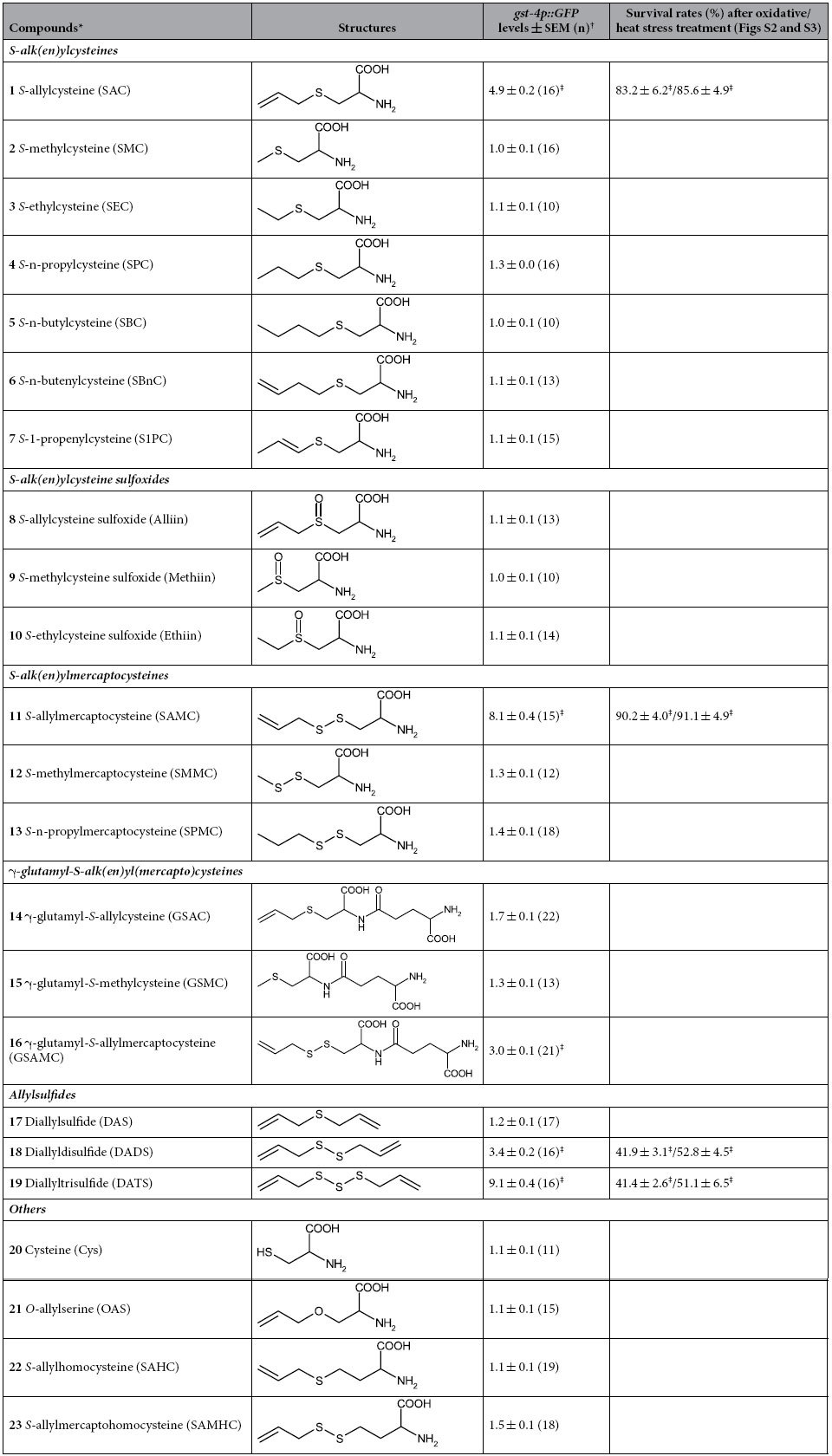
Relative *gst-4p::GFP* inducible activity and stress resistance capacity of garlic-derived organosulfur compounds and their analogs.

^*^Treated at 10 μM each for 24 hours at 20 °C. ^†^Relative fluorescence intensity with SEM. The number of animals tested in parentheses. ^‡^*P* < 0.001 vs control by one-way ANOVA with Tukey’s post hoc test.
