# Supplementary material for: Natural thioallyl compounds increase oxidative stress resistance and lifespan in Caenorhabditis elegans by modulating SKN-1/Nrf
Source: Sci Rep. 2016 Feb 22;6:21611. doi: 10.1038/srep21611 (PMC4761942; doi:10.1038/srep21611)
Supplement: Supplementary Information [file srep21611-s1.pdf]

Supplementary Information

Natural thioallyl compounds increase oxidative stress resistance and lifespan in *Caenorhabditis elegans* by modulating SKN-1/Nrf

Takahiro Ogawa[a,b,*], Yukihiro Kodera[b], Dai Hirata[a], T. Keith Blackwell[c], and Masaki Mizunuma[a,*]

[a] Department of Molecular Biotechnology, Graduate School of Advanced Sciences of Matter, Hiroshima University, Higashi-Hiroshima 739-8530, Japan
[b] Drug Discovery Laboratory, Wakunaga Pharmaceutical Co., Ltd, Hiroshima 739-1195, Japan
[c] Joslin Diabetes Center, Harvard Stem Cell Institute, and Harvard Medical School Department of Genetics, Boston, MA 02215, USA

[*] Correspondence to
Takahiro Ogawa (e-mail: ogawa_t@wakunaga.co.jp)
Masaki Mizunuma (email: mmizu49120@hiroshima-u.ac.jp)

Table of contents

Figure S1. Effect of SAC and SAMC on the proteasome activity……………………..…P2
Figure S2. Effect of OSCs on oxidative stress resistance………………….………..…P3
Figure S3. Effect of OSCs on heat stress resistance…………………………………..…P4
Table S1. Lifespans of wild-type shown in Figs. 1b and 1c………………………..….P5
Table S2. Lifespans of *daf-16(mgDf47)* shown in Figs. 2d and 2e……………….…...P6
Table S3. Lifespans of *skn-1(zu135)* shown in Figs. 3c and 3d…………………..……P7
Table S4. Nematode strains used in this study……………………………………..…..P8
Table S5. Primer sequences used in qRT-PCR analysis………………………………..P8
Supplementary Methods. 26S proteasome activity assays………………………..P9
Supplementary References……………………………………………………….P10

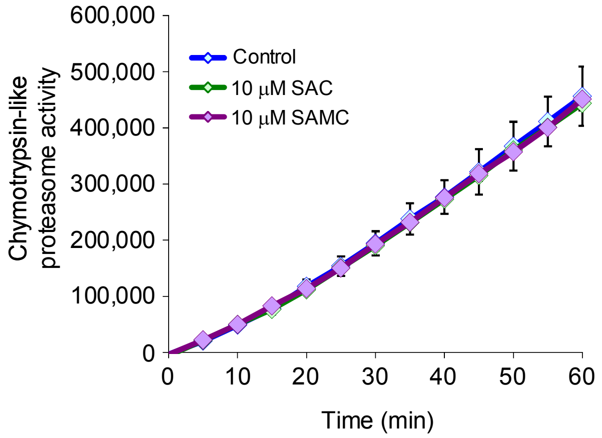

Figure S1. Effect of SAC and SAMC on the proteasome activity. The 26S proteasome activity in whole lysate (25 μg per sample) prepared using about 1,000 wild-type animals treated on the L1 stage with H_2O (control), SAC or SAMC for 4 days at 20ºC. Data represent mean ± SD (n = 3 of 1,000 animals).

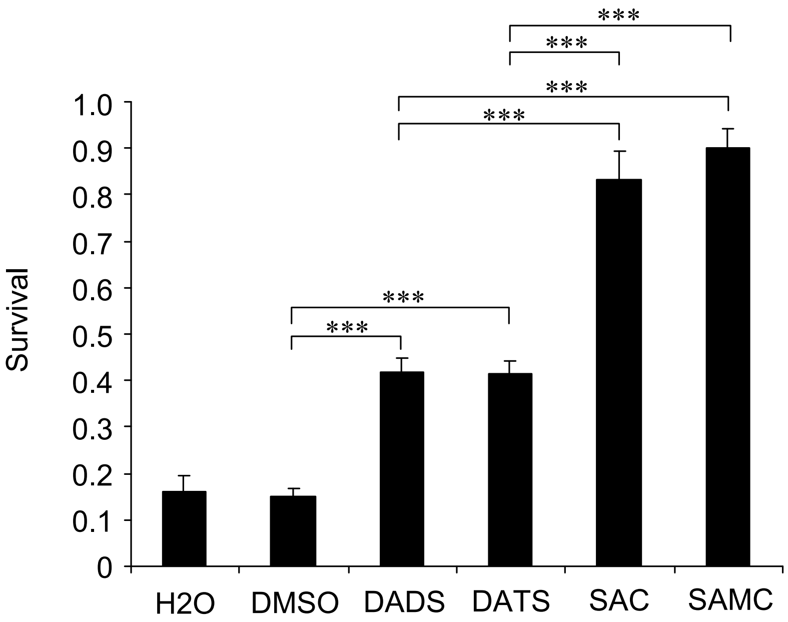

Figure S2. Effect of OSCs on oxidative stress resistance. Synchronized day-1 wild-type adults were treated with H_2O (control for SAC and SAMC), 0.02 % DMSO (control for DADS and DATS), DADS, DATS, SAC or SAMC for 48 hours at 20ºC and then subjected to oxidative stress (250 μM juglone for 2 hours at 20ºC). Each compound was treated at 10 μM. Survivals after the oxidative stress treatment were scored after a 16 hours recovery on NGM agar seeded with *E. coli* OP50. Data are represented as mean ± SD from three independent experiments. Total number of animals tested (H_2O, n=185; DMSO, n=199; DADS, n=198; DATS, n=200; SAC, n=217; SAMC, n=242). ***$P<0.001$ (one-way ANOVA with Tukey's post hoc test).

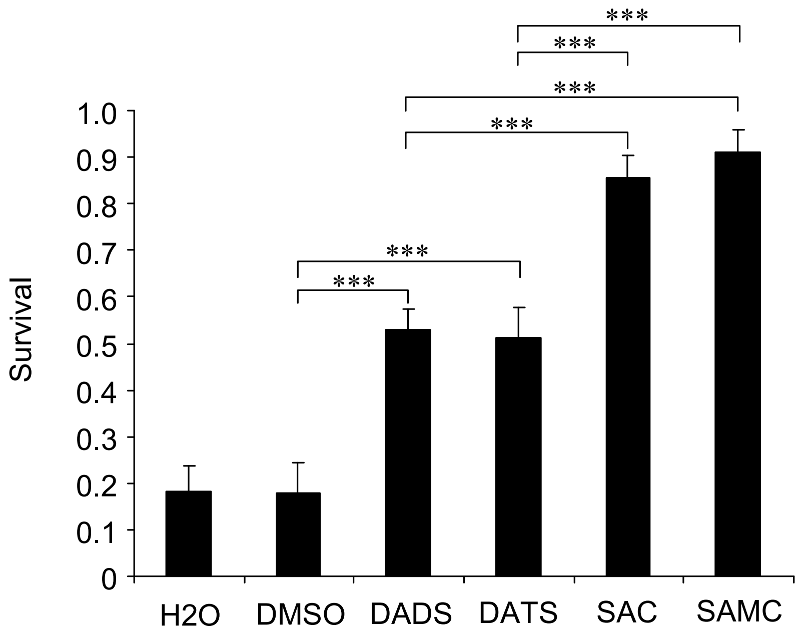

Figure S3. Effect of OSCs on heat stress resistance. Synchronized day-1 wild-type adults were treated with H_2O (control for SAC and SAMC), 0.02 % DMSO (control for DADS and DATS), DADS, DATS, SAC or SAMC for 48 hours at 20ºC and then subjected to heat stress (35ºC for 7 hours). Each compound was treated at 10 μM. Survivals after the oxidative stress treatment were scored after a 16 hours recovery on NGM agar seeded with *E. coli* OP50. Data are represented as mean ± SD from three independent experiments. Total number of animals tested (H_2O, n=173; DMSO, n=166; DADS, n=194; DATS, n=211; SAC, n=231; SAMC, n=224). ***P<0.001 (one-way ANOVA with Tukey's post hoc test).

Table S1. Lifespans of wild-type shown in Figs. 1b and 1c.

Trial	Treatment (μM)	No. animals	Censored	Mean lifespan [1)]			Maximum lifespan [2)]	
				days ± SEM	% extension	*P* value vs. control [3)]	days ± SEM	*P* value vs. control [4)]
1st	Control	81	3	20.9 ± 0.6	N/A	N/A	35	
	SAC (1)	85	4	22.3 ± 0.5	6.5%	0.171	32	
	SAC (10)	80	3	23.8 ± 0.6	13.8%	0.003	48	
	SAC (100)	70	9	23.8 ± 0.6	13.5%	0.001	37	
2nd	Control	106	4	21.6 ± 0.4	N/A	N/A	32	
	SAC (1)	100	2	23.1 ± 0.4	7.3%	0.080	37	
	SAC (10)	83	5	24.1 ± 0.4	11.6%	0.001	37	
	SAC (100)	92	11	23.7 ± 0.4	10.1%	0.007	32	
	SAMC (1)	94	3	22.8 ± 0.4	5.7%	0.136	32	
	SAMC (10)	94	11	25.1 ± 0.5	16.4%	<0.001	43	
	SAMC (100)	90	10	24.9 ± 0.5	15.3%	<0.001	42	
3rd	Control	54	0	21.9 ± 0.7	N/A	N/A	35	
	SAC (1)	61	2	23.6 ± 0.6	7.7%	0.196	31	
	SAC (10)	62	7	26.2 ± 0.7	20.0%	<0.001	50	
	SAC (100)	56	7	26.1 ± 0.5	19.3%	0.002	34	
	SAMC (1)	69	3	23.4 ± 0.6	7.1%	0.262	46	
	SAMC (10)	68	3	25.5 ± 0.8	16.6%	0.002	58	
	SAMC (100)	60	5	24.9 ± 0.6	13.9%	0.012	33	
4th	Control	53	0	20.7 ± 0.7	N/A	N/A	37	
	SAC (1)	49	2	22.5 ± 0.8	8.9%	0.159	35	
	SAC (10)	59	0	26.2 ± 0.6	26.7%	<0.001	33	
	SAC (100)	61	3	25.4 ± 0.7	23.2%	<0.001	35	
	SAMC (1)	50	2	21.5 ± 0.7	4.1%	0.521	30	
	SAMC (10)	58	4	26.1 ± 0.6	26.1%	<0.001	37	
	SAMC (100)	63	3	27.2 ± 0.6	31.6%	<0.001	37	
5th	Control	49	2	21.3 ± 0.6	N/A	N/A	32	
	SAMC (1)	59	1	21.9 ± 0.6	2.7%	0.683	31	
	SAMC (10)	54	5	25.6 ± 0.7	20.0%	<0.001	38	
	SAMC (100)	55	6	26.3 ± 0.7	23.5%	<0.001	38	
Combined	Control	343	9	21.3 ± 0.3	N/A	N/A	34.8 ± 1.0	N/A
(Trial 1~5)	SAC (1)	295	10	22.9 ± 0.3	7.5%	<0.001	33.8 ± 1.4	0.989
Fig. 1b and c	SAC (10)	284	15	24.9 ± 0.3	17.0%	<0.001	42.0 ± 4.1	0.171
	SAC (100)	279	30	24.6 ± 0.3	15.6%	<0.001	34.5 ± 1.0	0.999
	SAMC (1)	272	9	22.5 ± 0.3	5.8%	0.024	34.8 ± 3.8	1.000
	SAMC (10)	274	23	25.5 ± 0.3	19.7%	<0.001	44.0 ± 4.8	0.236
	SAMC (100)	268	24	25.7 ± 0.3	20.9%	<0.001	37.5 ± 1.8	0.971

1) Mean lifespan is the day when 50% of worms survived.

2) Maximum lifespan is the day when the last surviving worm died.

3) *P*-values for mean lifespan were obtained by log-rank test by comparing the control and other treated groups.

4) *P*-values for maximum lifespan were calculated by one-way ANOVA with Tukey's post hoc test from means of the maximum lifespan of each condition.

Table S2. Lifespans of *daf-16(mgDf47)* shown in Figs. 2d and 2e.

Trial	Treatment (μM)	No. animals	Censored	Mean lifespan [1)] days ± SEM	% extension	*P* value vs. control [3)]	Maximum lifespan [2)] days ± SEM	*P* value vs. control [4)]
1st	Control	41	2	12.3 ± 0.5	N/A	N/A	18	
	SAC (1)	41	2	12.8 ± 0.4	4.2%	0.873	18	
	SAC (10)	42	1	13.8 ± 0.3	12.2%	0.089	20	
	SAC (100)	41	0	13.9 ± 0.3	13.4%	0.048	20	
	SAMC (1)	42	3	12.4 ± 0.5	1.3%	0.719	20	
	SAMC (10)	41	3	12.8 ± 0.5	4.8%	0.537	22	
	SAMC (100)	42	3	13.2 ± 0.3	7.7%	0.368	18	
2nd	Control	80	0	11.7 ± 0.4	N/A	N/A	18	
	SAC (1)	79	0	12.2 ± 0.4	4.3%	0.551	21	
	SAC (10)	75	0	13.4 ± 0.4	14.2%	0.008	18	
	SAC (100)	80	4	12.7 ± 0.4	8.2%	0.148	19	
	SAMC (1)	85	0	11.4 ± 0.4	-3.1%	0.501	18	
	SAMC (10)	81	1	13.0 ± 0.3	10.6%	0.081	20	
	SAMC (100)	80	1	13.1 ± 0.3	11.6%	0.039	19	
3rd	Control	69	0	11.4 ± 0.4	N/A	N/A	20	
	SAC (1)	66	0	11.3 ± 0.4	-0.6%	0.607	18	
	SAC (10)	68	2	12.7 ± 0.4	11.2%	0.061	22	
	SAC (100)	70	3	13.7 ± 0.4	20.4%	<0.001	22	
	SAMC (1)	75	0	11.4 ± 0.4	-0.3%	0.578	22	
	SAMC (10)	86	0	13.1 ± 0.3	15.3%	0.004	21	
	SAMC (100)	80	3	13.8 ± 0.4	21.5%	<0.001	23	
Combined	Control	190	2	11.7 ± 0.3	N/A	N/A	18.7 ± 0.7	N/A
(Trial 1~3)	SAC (1)	186	2	12.0 ± 0.2	2.6%	0.756	19.0 ± 1.0	0.994
Fig. 2d and e	SAC (10)	185	3	13.2 ± 0.2	12.8%	<0.001	20.0 ± 1.2	0.754
	SAC (100)	191	7	13.3 ± 0.2	13.8%	<0.001	20.3 ± 0.9	0.616
	SAMC (1)	202	3	11.6 ± 0.2	-1.2%	0.481	20.0 ± 1.2	0.808
	SAMC (10)	208	4	13.0 ± 0.2	11.1%	0.002	21.0 ± 0.6	0.447
	SAMC (100)	202	7	13.4 ± 0.2	14.5%	<0.001	20.0 ± 1.5	0.808

1) Mean lifespan is the day when 50% of worms survived.

2) Maximum lifespan is the day when the last surviving worm died.

3) *P*-values for mean lifespan were obtained by log-rank test by comparing the control and other treated groups.

4) *P*-values for maximum lifespan were calculated by one-way ANOVA with Tukey's post hoc test from means of the maximum lifespan of each condition.

Table S3. Lifespans of *skn-1(zu135)* shown in Figs. 3c and 3d.

Trial	Treatment (μM)	No. animals	Censored	Mean lifespan [1]			Maximum lifespan [2]	
				days ± SEM	% extension	*P* value vs. control [3]	days ± SEM	*P* value vs. control [4]
1st	Control	32	0	17.3 ± 1.1	N/A	N/A	37	
	SAC (10)	32	0	18.0 ± 1.1	3.6%	0.779	34	
	SAC (100)	32	1	16.4 ± 0.8	-5.7%	0.405	27	
	SAMC (10)	32	0	18.5 ± 1.4	6.7%	0.469	39	
	SAMC (100)	32	2	15.8 ± 1.0	-9.1%	0.337	28	
2nd	Control	49	0	15.9 ± 0.7	N/A	N/A	28	
	SAC (10)	50	1	16.7 ± 0.8	5.0%	0.409	37	
	SAC (100)	53	1	14.5 ± 0.5	-9.1%	0.041	23	
	SAMC (10)	51	0	16.4 ± 0.9	2.6%	0.704	37	
	SAMC (100)	52	0	13.9 ± 0.6	-12.8%	0.020	28	
3rd	Control	59	0	18.5 ± 0.6	N/A	N/A	36	
	SAC (10)	53	4	17.8 ± 0.6	-3.9%	0.525	32	
	SAC (100)	57	6	15.9 ± 0.5	-13.8%	0.002	27	
	SAMC (10)	54	3	17.2 ± 0.6	-7.1%	0.137	34	
	SAMC (100)	56	1	16.1 ± 0.5	-12.7%	0.004	27	
Combined	Control	140	0	17.3 ± 0.4	N/A	N/A	33.7 ± 2.8	N/A
(Trial 1~3)	SAC (10)	135	5	17.4 ± 0.5	0.5%	0.853	34.3 ± 1.5	0.970
Fig. 3c and d	SAC (100)	142	8	15.5 ± 0.3	-10.8%	<0.001	25.7 ± 1.3	0.067
	SAMC (10)	137	3	17.2 ± 0.5	0.9%	0.855	36.7 ± 1.5	0.525
	SAMC (100)	140	3	15.2 ± 0.4	-12.3%	<0.001	27.7 ± 0.3	0.134

1) Mean lifespan is the day when 50% of worms survived.

2) Maximum lifespan is the day when the last surviving worm died.

3) *P*-values for mean lifespan were obtained by log-rank test by comparing the control and other treated groups.

4) *P*-values for maximum lifespan were calculated by one-way ANOVA with Tukey's post hoc test from means of the maximum lifespan of each condition.

Table S4. Nematode strains used in this study.

Number	Genetic background	Transgene	Array number	Referenced
N2	Wild-type			
CF1553	N2	Is[*sod-3p::GFP*]		(1) Libina *et al.*, 2003
CL2070	N2	Is[*hsp-16.2p::GFP*]		(2) Link *et al.*, 1999
CL2166	N2	Is[*gst-4p::GFP*]		(3) Link and Johnson, 2002
	daf-16(mgDf47)			(4) Ogg *et al.*, 1997
LD1482	*daf-16(mu86)*	Is[*DAF-16A::GFP*]		(5) Lin *et al.*, 2001
CL691	*skn-1(zu67)*	Is[*gst-4p::GFP*]		(6) Rea *et al.*, 2007
EU31	*skn-1(zu135)*			(7) Bowerman *et al.*, 1992
LD001	N2	Is[*SKN-1B/C::GFP*]	007	(8) An & Blackwell, 2003
KU4	*sek-1(km4)*			(9) Tanaka-Hino *et al.*, 2002
NL2099	*rrf-3(pk1426)*			(10) Simmer *et al.*, 2002

Table S5. Primer sequences used in qRT-PCR analysis.

Gene name	Forward primer sequence (5'-3')	Reverse primer sequence (5'-3')
act-1	ACCATGTACCCAGGAATTGC	TGGAAGGTGGAGAGGGAAG
sod-3	AGCATCATGCCACCTACGTGA	CACCACCATTGAATTTCAGCG
hsp-16.2	CTCAACGTTCCGTTTTTGGT	CGTTGAGATTGATGGCAAAC
ctl-2	TCCGTGACCCTATCCACTTC	TGGGATCCGTATCCATTCAT
gst-4	CGTTTTCTATGGAAGTGACGC	TCAGCCCAAGTCAATGAGTC
gcs-1	TGTTGATGTGGATACTCGGTG	TGTATGCAGGATGAGATTGTACG
gst-10	GTCTACCACGTTTTGGATGC	ACTTTGTCGGCCTTTCTCTT
atf-5	CCATCAATCTTATCAACAGCATCAT	CTGGTGGAACCGAAGTG
haf-7	GACGTGGAAAAGCTGAGAGG	GCAGGGAAAATGTGAGGAAA
rpt-3	CCCAAGAGGAGTTCTCATGTA	ATGAAGGAAGCAGCAGTATT
rpn-12	CTGCCAACAGATTGTCCG	GGCGTAGAGATGTAAGCG
pas-4	CGAGCCATCTGGAGCTTACTA	TCCTCAAGGTATTCACGCAC
pbs-6	TGGACAGAGCCATCTCATT	CTTCAGCGATGACCAAGTG
skn-1	AGTGTCGGCGTTCCAGATTTC	GTCGACGAATCTTGCGAATCA

Supplementary Methods

26S proteasome activity assays

The 26S proteasome activity in whole animal lysate was measured as previously described (11). Briefly, after treating L1 larvae with H_2O (control), SAC or SAMC (10 µM each) for 4 days at 20ºC, adult animals were sonicated in 4 volumes of lysis buffer (50 mM Tris-HCl, pH 7.5, 250 mM sucrose, 5 mM $MgCl_2$, 2 mM ATP, 1 mM dithiothreitol and 0.5 mM EDTA) with a Bioruptor UCW310 (BM Equipment, Tokyo, Japan). Lysate was centrifuged at 14,000 X g for 10 min at 4ºC. To measure chymotrypsin-like proteasome activity, 25 µg of whole animal lysate was transferred to a 96-well microtitre plate, then incubated with a fluorogenic peptide substrate (100 µM Suc-Leu-Leu-Val-Tyr-AMC, Boston Biochemicals, MA) in proteasome activity assay buffer (50 mM Tris-HCl, pH 7.5, 40 mM KCl, 5 mM $MgCl_2$, 0.5 mM ATP, 1 mM dithiothreitol and 0.05 mg mL^{-1} BSA) at 25ºC. The fluorescence intensity was measured at 380 nm for excitation and 460 nm for emission using an EnVision 2104 multilabel reader (PerkinElmer, Waltham, MA) every 5 min for 1 hour at 25ºC. The assay was performed in the absence or presence of proteasome inhibitor (40 µM Epoxomicin, Peptide Institute, Osaka, Japan) to calculate the 26S proteasome-specific activity.

Supplementary References

(1) Libina, N., Berman, J. R. & Kenyon, C. Tissue-specific activities of *C. elegans* DAF-16 in the regulation of lifespan. *Cell* **115,** 489-502 (2003).

(2) Link, C. D., Cypser, J. R., Johnson, C. J. & Johnson, T. E. Direct observation of stress response in *Caenorhabditis elegans* using a reporter transgene. *Cell Stress Chaperones* **4,** 235-242 (1999).

(3) Link, C. D. & Johnson, C. J. Reporter transgenes for study of oxidant stress in *Caenorhabditis elegans*. *Methods Enzymol.* **353,** 497-505 (2002).

(4) Ogg, S. *et al*. The Fork head transcription factor DAF-16 transduces insulin-like metabolic and longevity signals in *C. elegans*. *Nature* **389,** 994-999 (1997).

(5) Lin, K., Hsin, H., Libina, N. & Kenyon, C. Regulation of the *Caenorhabditis elegans* longevity protein DAF-16 by insulin/IGF-1 and germline signaling. *Nat. Genet.* **28,** 139-145 (2001).

(6) Rea, S. L., Ventura, N. & Johnson, T. E. Relationship between mitochondrial electron transport chain dysfunction, development, and life extension in *Caenorhabditis elegans*. *PLoS Biol.* **5,** e259 (2007).

(7) Bowerman, B., Eaton, B. A. & Priess, J. R. *skn-1*, a maternally expressed gene required to specify the fate of ventral blastomeres in the early *C. elegans* embryo. *Cell* **68,** 1061-1075 (1992).

(8) An, J. H. & Blackwell, T. K. SKN-1 links *C. elegans* mesendodermal specification to a conserved oxidative stress response. *Genes dev.* **17,** 1882-1893 (2003).

(9) Tanaka-Hino, M. *et al*. SEK-1 MAPKK mediates Ca^{2+} signaling to determine neuronal asymmetric development in *Caenorhabditis elegans*. *EMBO Rep.* **3,** 56-62 (2002).

(10) Simmer, F. *et al*. Loss of the putative RNA-directed RNA polymerase RRF-3 makes *C. elegans* hypersensitive to RNAi. *Curr. Biol.* **12,** 1317-1319 (2002).

(11) Kisselev, A. F. & Goldberg, A. L. Monitoring activity and inhibition of 26S proteasomes with fluorogenic peptide substrates. *Methods Enzymol.* **398,** 364-378 (2005).
